# Time series-based forecasting of infectious disease outbreak using information systems in public health

**DOI:** 10.3389/fpubh.2025.1680534

**Published:** 2026-05-01

**Authors:** Mingyu Du

**Affiliations:** Medical School, Shandong Jiaotong University, Jinan, China

**Keywords:** epidemic forecasting, spatial-temporal attention, causal regularization with semantic anchors, domain knowledge integration, interpretability in neural models

## Abstract

**Introduction:**

The escalating frequency of infectious disease outbreaks underscores the urgent need for reliable forecasting systems to support timely public health interventions. Existing approaches—ranging from statistical heuristics to black-box deep learning models—often lack domain awareness, adaptability, and interpretability, limiting their utility in dynamic outbreak scenarios.

**Methods:**

This study proposes EpiCastNet, a forecasting framework that integrates spatiotemporal attention mechanisms with a hybrid encoding architecture to jointly model empirical patterns and rule-based constraints. A key component is the Causal Regularization with Semantic Anchors (CRSA) module, which incorporates epidemiological principles, such as intervention efficacy and seasonal transmission dynamics, into the model's differentiable training process. This enhances both semantic alignment and robustness under real-world uncertainties.

**Results and discussion:**

Empirical evaluations on public health time-series datasets, including COVIDcast and JHU COVID-19, demonstrate that EpiCastNet consistently outperforms state-of-the-art methods in terms of RMSE, MAE, R^2^, and MAPE, while maintaining high stability under noisy and incomplete data conditions. These findings highlight the framework's effectiveness and interpretability in epidemic forecasting, offering a practical tool for data-driven decision-making in public health surveillance.

## 1 Introduction

The rapid spread of infectious diseases poses a significant threat to global public health, necessitating timely and accurate forecasting to support effective intervention strategies. Traditional surveillance methods often struggle to provide real-time insights, leading to delayed responses and exacerbated outbreaks (1). Consequently, the integration of information systems into public health infrastructure has become critical. These systems not only facilitate efficient data collection and sharing but also enable the development of predictive models capable of forecasting disease dynamics (2). The use of time series forecasting in this context is particularly compelling, as it leverages historical data to identify trends and patterns, supporting proactive public health measures (3). Moreover, the emergence of digital health records, sensor networks, and mobile data has enriched the quality and granularity of available data, further enhancing forecasting capabilities (4). In light of these developments, understanding the evolution of forecasting methods within information systems is crucial to advancing public health responses.

Early methods for infectious disease forecasting primarily relied on structured frameworks that captured domain-specific knowledge. These approaches utilized predefined rules and logical constructs to model disease characteristics such as incubation periods, transmission rates, and environmental factors (5). Although providing clarity and explainability, such frameworks encountered difficulties in accommodating the evolving patterns of disease development and the intricate characteristics of practical datasets (6). The manual effort required to encode domain expertise into actionable models also hindered scalability. Furthermore, these methods often lacked the flexibility to incorporate emerging patterns, limiting their effectiveness in responding to new outbreaks (7). As a result, researchers sought alternative approaches that could better accommodate variability and complexity.

In response to the limitations of rigid frameworks, researchers began employing flexible computational techniques that could extract predictive patterns directly from data. These methods leveraged statistical algorithms to analyze temporal correlations and identify trends within time series datasets (8). By incorporating features such as seasonality indicators and lagged variables, these models improved the ability to forecast disease dynamics (9). Ensemble methods further enhanced robustness by combining multiple predictive models. Despite these advancements, the reliance on handcrafted features constrained their ability to fully capture intricate temporal relationships (10). Additionally, many models faced challenges in handling sequential dependencies, which are critical for understanding long-term interactions in disease transmission.

The emergence of sophisticated deep learning frameworks has represented a pivotal advancement in capturing intricate time-based correlations. Approaches such as recursive architectures, attention-driven modules, and transformer-inspired designs have transformed epidemic prediction by extracting layered representations directly from unprocessed inputs (11). These strategies bypass traditional manual feature crafting, instead relying on automated inference to reveal nuanced interdependencies (12). Moreover, the fusion of diverse data modalities—including movement trajectories, environmental variables, and online behavioral signals—has enhanced the resilience and precision of predictive outputs (13). Although these algorithms often entail substantial computational overhead and pose challenges in terms of explainability, they remain at the forefront of forecasting capabilities within the public health domain (14). Their proficiency in managing complex, high-dimensional datasets and in modeling extended temporal links underscores their essential role in contemporary epidemiological modeling systems.

In light of the previously discussed constraints—particularly the limited transparency of deep learning algorithms and the rigidity of symbolic and conventional machine learning techniques—the study introduce a composite framework that synthesizes the advantages of all three methodologies. The strategy integrates symbolic inference for embedding domain-specific expertise, traditional learning models for structured pattern extraction, and deep architectures for modeling complex temporal behaviors. This fusion not only fulfills the requirements for explainability and resilience but also significantly improves predictive precision across varied epidemic contexts. Additionally, the modular design of the system enables flexible updates and scalability, essential for addressing the dynamic nature of public health landscapes. Through the deployment of an integrated information infrastructure that unites these approaches, the study strive to establish a more holistic and efficient paradigm for temporal prediction of infectious disease spread—capable of driving prompt and reliable intervention planning.

- The model introduces an integrated reasoning and learning framework, bridging rule-based knowledge with deep learning for enhanced adaptability and insight.
- It supports multi-source data fusion and robust temporal modeling, ensuring high accuracy and broad applicability across various disease scenarios.
- Empirical results show superior performance on benchmark datasets, achieving up to 15% improvement in forecasting accuracy over state-of-the-art baselines.

## 2 Related work

### 2.1 Statistical time series models

Historically, statistical models for temporal analysis have played a foundational role in epidemic forecasting within the realm of public health, offering techniques adept at uncovering time-dependent structures in disease-related datasets (4). Classical models like the Autoregressive Integrated Moving Average (ARIMA) and its seasonal extension (SARIMA) have proven valuable in detecting cyclical behaviors and linear progressions in infection rate records (11). Numerous investigations have employed these frameworks to anticipate future outbreaks, extrapolating from prior incidence data to model potential transmission trajectories (12). However, the effectiveness of these models is often constrained by their reliance on linearity and stationarity assumptions, which hinder their ability to accommodate the complex, nonlinear evolution of infectious diseases (13). To address these shortcomings, blended modeling strategies have emerged, combining ARIMA with machine learning algorithms to boost forecasting precision (15). For example, research has shown that combining ARIMA with Support Vector Machines or Neural Networks yields significant improvements in capturing nonlinearities inherent in disease datasets (14). These hybrid models benefit from the statistical rigor of ARIMA while utilizing machine learning techniques to address complex temporal relationships (16). Challenges remain in optimizing model parameters and ensuring the availability of high-resolution data for accurate predictions (17). Furthermore, the complexity of hybrid models can pose interpretability issues, particularly for public health practitioners who prioritize actionable insights (18). As such, while statistical time series models continue to be pivotal in epidemiological forecasting, ongoing advancements are required to improve their adaptability, transparency, and robustness in diverse public health applications.

### 2.2 Deep learning for epidemic forecasting

Advanced neural network techniques have become pivotal in analyzing the temporal behaviors of infectious disease spread, with models such as Recurrent Neural Networks (RNNs) and Long Short-Term Memory (LSTM) architectures showing strong proficiency in learning intricate patterns from sequential epidemiological data (19). These deep learning frameworks are particularly adept at managing nonlinear structures and capturing long-range temporal correlations, positioning them as highly effective for outbreak prediction tasks (20). LSTM models, in particular, have been successfully employed to estimate infection counts for illnesses such as COVID-19, consistently surpassing conventional statistical approaches in forecasting performance (21). Their strength lies in their ability to preserve context over prolonged time intervals, which enables them to reflect the nuanced and evolving nature of disease transmission dynamics (22). Innovations such as attention mechanisms have further improved the performance of deep learning models by enabling focused analysis of relevant data segments, thus refining forecast precision (23). Transfer learning techniques have also facilitated the application of models trained on well-documented regions to areas with limited data availability, addressing disparities in data accessibility (24). Nonetheless, these models often require extensive datasets for training, which can be a barrier in resource-constrained settings (25). Additionally, the opacity of deep learning models, often described as “black box" systems, raises concerns about their interpretability and acceptance in public health decision-making contexts (26). Efforts to enhance transparency through explainable AI methodologies are underway, aiming to elucidate the reasoning behind model predictions (27). The computational demands associated with training deep learning models necessitate access to advanced computing infrastructure, posing logistical challenges for widespread implementation (28). Therefore, while deep learning offers significant promise for epidemic forecasting, addressing issues related to data requirements, interpretability, and computational resources remains essential for its effective integration into public health practices.

Deep learning has emerged as a powerful tool for epidemic forecasting due to its ability to capture complex temporal dynamics, non-linear relationships, and multi-source data dependencies. Among the earliest approaches, Recurrent Neural Networks (RNNs) and their variants such as Long Short-Term Memory (LSTM) networks demonstrated strong performance in modeling sequential epidemic data, particularly in short-term forecasting. However, these models often struggle with vanishing gradients and limited long-term memory. To address these issues, Transformer-based models have gained prominence in time series forecasting. Informer ?, Autoformer ?, and TimesNet ? introduced efficient self-attention mechanisms and decomposition strategies to model long-range temporal dependencies. These models have shown strong performance on benchmark datasets, but often lack domain-specific interpretability and require large volumes of data for effective training. PatchTST ? further improved locality modeling via patch-level learning, which is useful for capturing regional temporal features in epidemic data. More recently, hybrid models combining graph neural networks (GNNs) and temporal encoders have been proposed for spatially-aware forecasting. For example, He et al. (29) use GNNs to trace disease source nodes in dynamic contact networks. Such approaches effectively capture inter-regional transmission but may require accurate mobility or contact graph data, which is not always available. Another emerging direction is logic-informed or causal-regularized neural forecasting, where domain knowledge (such as seasonality, interventions) is encoded into the training process. Li et al. (30) has demonstrated how embedding semantic constraints can improve both accuracy and interpretability. However, most existing approaches apply rigid constraints or static rules, limiting generalization across contexts. The proposed method builds upon these foundations by combining deep temporal encoding, graph-based spatial modeling, and a flexible causal regularization framework. Unlike prior works that treat forecasting as a pure black-box task, this approach aligns predictive modeling with public health semantics, offering enhanced robustness and decision utility in epidemic scenarios.

### 2.3 Integration of information systems in public health surveillance

The integration of information systems into public health surveillance has profoundly transformed the capacity to monitor and predict infectious disease outbreaks, leveraging diverse data sources to generate actionable insights in real time (31). Modern surveillance frameworks utilize electronic health records, laboratory test results, and syndromic data to construct comprehensive views of disease trends (32). The application of advanced analytics and machine learning algorithms has enabled the detection of anomalies and early warning signals, facilitating prompt interventions by public health authorities (11). Digital platforms and mobile applications have further enhanced the efficiency of data collection and analysis, improving the accuracy and timeliness of epidemiological forecasts (12). Geographic Information Systems (GIS) have played a pivotal role in mapping disease spread, allowing for spatial analysis and resource allocation based on identified hotspots (13). The COVID-19 pandemic highlighted the critical importance of integrated surveillance infrastructures, as nations equipped with robust systems were better positioned to track and respond to dynamic outbreak scenarios (15). However, challenges persist in ensuring the interoperability of disparate systems and maintaining the quality of collected data (14). Standardizing data formats and establishing protocols for secure data sharing are necessary to maximize the utility of integrated systems (16). Furthermore, the development of user-friendly interfaces and visualization tools can aid public health officials in interpreting complex datasets, thereby supporting evidence-based decision-making (17). As the landscape of infectious disease threats continues to evolve, the advancement of information systems remains a cornerstone in enabling proactive and coordinated public health responses (18).

Recent literature published in 2025 has continued to expand the landscape of epidemic forecasting. Babanejaddehaki et al. (33) provide a comprehensive survey of outbreak detection and forecasting methods, covering classical statistical models, machine learning techniques, and hybrid approaches, along with diverse data sources from clinical, behavioral, and environmental domains. He et al. (29) propose a graph neural network-based framework for tracing epidemic sources in dynamic contact networks, which supports the integration of spatial structures in predictive modeling. Li et al. (30) explore the influence of individual psychological security and information availability on infectious disease spread, offering a novel direction that complements traditional epidemiological variables. In addition, Du et al. (34) investigate aerosol transmission dynamics in livestock, which, although focused on animal health, provide physical insights that may inform future extensions of human-pathogen forecasting frameworks. These recent developments further underscore the importance of embedding causal reasoning, behavioral signals, and graph-based dependencies into modern epidemic forecasting systems.

## 3 Method

### 3.1 Overview

This section outlines the methodological framework for advancing public health forecasting, a domain where predictive models play a pivotal role in informing policy decisions, optimizing resource distribution, and responding to epidemics. The proposed approach systematically addresses critical challenges, including the handling of temporal dependencies, the heterogeneity inherent in spatial and demographic data, and the integration of expert knowledge within data-driven systems. The framework is structured to provide a comprehensive solution by combining theoretical rigor with practical innovations.

The public health forecasting problem is first formalized to establish a clear mathematical foundation. Time-evolving health indicators are represented using structured notations, capturing both observed surveillance data and latent dynamics that govern health outcomes. The objective is defined as a forecasting task that incorporates domain-specific priors alongside observed data streams. This formalization ensures alignment with existing literature while providing a unified basis for the subsequent methodological contributions. The study introduce the novel forecasting architecture, *EpiCastNet*, which employs a hybrid design integrating temporal deep learning with symbolic knowledge regularization. The proposed architecture effectively models both short-term fluctuations and prolonged epidemiological developments by adaptively focusing on organized inputs such as climatic variables, medical infrastructure availability, and population behavior dynamics. A central innovation lies in its mixed-representation module, which fuses neural-derived features with rule-based constraints informed by domain-specific health knowledge frameworks. Furthermore, the design integrates a structured memory component that preserves and utilizes pivotal disease-related occurrences, thereby improving the model's capacity to generalize across heterogeneous geographic and contextual settings.

To further refine the learning process, the study propose *Causal Regularization with Semantic Anchors* (CRSA), a strategy that introduces domain-oriented constraints as soft differentiable objectives. These constraints encode expert knowledge in the form of logical relationships, such as the influence of vaccination on infection rates or the role of mobility in transmission dynamics. The regularization approach balances fidelity to training data with adherence to these semantic rules, resulting in forecasts that are both interpretable and robust across various scenarios. The modularity of the framework facilitates the integration of future epidemiological insights, and its generality extends its applicability to a wide range of forecasting tasks, including those addressing both infectious and non-communicable diseases. The efficacy of the framework is demonstrated through extensive experiments on real-world datasets, with results elaborated in subsequent sections.

The forecasting task addressed in this study focuses on predicting epidemiological indicators such as new case counts or disease incidence over future time steps, based on historical observations and auxiliary features. Formally, given a multivariate time series consisting of health indicators $Y_{t}\in {\mathbb{R}}^{d_{y}}$ and contextual features $X_{t}\in {\mathbb{R}}^{d_{x}}$, the objective is to estimate future values Ŷ_*T*+1:*T*+*H*_ for a predefined prediction horizon *H*. The forecasting is performed at regular temporal intervals (such as daily or weekly), and the model operates at region-specific resolutions, enabling both local and global predictions. Unlike conventional sequence models that treat inputs as homogeneous vectors, the proposed framework decomposes the forecasting problem into spatial, temporal, and semantic components. The temporal encoder captures both short-term and long-range dependencies through gated recurrent layers augmented with attention mechanisms. The spatial module leverages inter-regional mobility or adjacency information to capture geographically distributed transmission patterns. Domain knowledge is injected via logic-aware semantic constraints that encode assumptions such as seasonality, intervention effects, and causal links (such as vaccination reduces incidence). The model not only outputs point estimates but also provides probabilistic forecasts by predicting both the mean and variance of future values, which allows for uncertainty quantification. This is particularly important in public health settings, where robust decision-making often depends on confidence intervals and worst-case scenarios. The inclusion of a Kullback-Leibler divergence term regularizes the uncertainty modeling, ensuring consistency and stability in the predictive distribution. By structuring the forecasting process around real epidemiological signals and enhancing it with multi-dimensional modeling and expert-informed constraints, the proposed method is well-aligned with the title and objective of the study. The detailed architectural breakdown and experimental validation demonstrate the model's strength in practical disease forecasting applications.

### 3.2 Preliminaries

The study address the problem of public health forecasting over a discrete time horizon, aiming to predict future values of epidemiological indicators based on historical observations and auxiliary covariates. This section provides a mathematical formulation of the problem, introduces the notation used throughout the study, and outlines the primary assumptions underlying the proposed methodology.

Let T={1,2,…,T} represent the set of time indices for which data is available, and let $Y={\left\{Y_{t}\right\}}_{t\in T}$ denote the temporal sequence of target public health variables. Each $Y_{t}\in {\mathbb{R}}^{d_{y}}$ corresponds to observed indicators at time *t*, where *d*_*y*_ is the dimensionality of the health outcomes being tracked. The forecasting task is to estimate future values Ŷ_*T*+τ_ for τ∈{1, …, *H*}, where *H* denotes the prediction horizon.

The model inputs consist of a sequence of observed covariates $X={\left\{X_{t}\right\}}_{t\in T}$, with each $X_{t}\in {\mathbb{R}}^{d_{x}}$ representing contextual features. These covariates may include environmental data, demographic attributes, mobility patterns, vaccination statistics, and other relevant factors. The generative structure of the observations is expressed as:


$$\begin{array}{l} Y_{t}=f^{*}\left(X_{\leq t},Y_{<t},Z_{t},\epsilon_{t}\right),\;\forall t\in T, \end{array}$$ (1)

where f^*^ is an unknown mapping that may exhibit non-linear and temporally non-stationary behavior. The term *Z*_*t*_ denotes latent exogenous factors such as pathogen characteristics or healthcare system disruptions, and ϵ_*t*_ is a stochastic noise term.

To approximate f^*^, the study define a parameterized function class F, where $f_{\theta }\in F$ depends on parameters θ. The predictive model operates over a sliding window of past observations:


$$\begin{array}{l} f_{\theta }:\left(X_{t-w+1:t},Y_{t-w+1:t-1}\right)\mapsto {Ŷ}_{t}, \end{array}$$ (2)

where *w* specifies the window size. The optimal parameters θ^*^ are determined by minimizing a forecasting error criterion $L_{\text{forecast}}$:


$$\begin{array}{l} \theta^{*}=\arg\underset{\theta \in \Theta }{\min}\overset{T}{\underset{t=w+1}{\sum }}L_{\text{forecast}}\left(Y_{t},f_{\theta }\left(X_{t-w+1:t},Y_{t-w+1:t-1}\right)\right). \end{array}$$ (3)

Public health dynamics exhibit dependencies across temporal, spatial, and structural dimensions. To capture spatial dependencies, the study define a domain $R=\left\{r_{1},r_{2},\ldots ,r_{M}\right\}$ representing *M* administrative units. The outcome variable becomes $Y_{t}^{\left(r\right)}$ and the covariates $X_{t}^{\left(r\right)}$ for each region r∈R:


$$\begin{array}{l} Y_{t}^{\left(r\right)}=f_{\theta }\left(\left\{X_{t-w+1:t}^{\left(r\right)},Y_{t-w+1:t-1}^{\left(r\right)}\right\},C_{t}^{\left(r\right)}\right), \end{array}$$ (4)

where $C_{t}^{\left(r\right)}$ represents context derived from neighboring or hierarchical regions.

A graph G=(R,E) is used to encode spatiotemporal correlations, where $\left(r_{i},r_{j}\right)\in E$ indicates epidemiological interactions such as human mobility or resource sharing. Let *A*∈{0, 1}^*M*×*M*^ denote the adjacency matrix. Regional features are updated using graph-based aggregation:


$$\begin{array}{l} {\tilde{X}}_{t}^{\left(r\right)}=\underset{r^{\prime }\in N\left(r\right)}{\sum }A_{rr^{\prime }}\cdot X_{t}^{\left(r^{\prime }\right)},\;{Ỹ}_{t}^{\left(r\right)}=\underset{r^{\prime }\in N\left(r\right)}{\sum }A_{rr^{\prime }}\cdot Y_{t}^{\left(r^{\prime }\right)}, \end{array}$$ (5)

where N(r) represents the neighborhood of region *r*.

Health indicators are influenced by latent structured constraints such as seasonality, causality, or intervention effects. Let K denote a set of domain-specific constraints encoded symbolically. Each k∈K imposes logical conditions on the predicted outputs:


$$\begin{array}{l} k\left({Ŷ}_{T+1:T+H}\right)=\text{True}. \end{array}$$ (6)

To enforce these constraints, a semantic regularization term $L_{\text{sem}}$ is introduced, penalizing violations:


$$\begin{array}{l} L_{\text{sem}}=\underset{k\in K}{\sum }I\left[k\left({Ŷ}_{T+1:T+H}\right)=\text{False}\right]\cdot \rho \left(k\right), \end{array}$$ (7)

where ρ(*k*) is a penalty weight reflecting domain-prior confidence.

Dynamic attention mechanisms are incorporated to adaptively weight predictive signals. Let $\alpha_{t}^{\left(j\right)}$ denote the attention weight for the *j*th feature at time *t*:


$$\begin{array}{l} \alpha_{t}^{\left(j\right)}=\frac{\exp\left(h^{⊤}\tanh\left(W_{x}X_{t}^{\left(j\right)}+W_{y}Y_{t-1}^{\left(j\right)}\right)\right)}{\overset{d_{x}}{\underset{j^{\prime }=1}{\sum }}\exp\left(h^{⊤}\tanh\left(W_{x}X_{t}^{\left(j^{\prime }\right)}+W_{y}Y_{t-1}^{\left(j^{\prime }\right)}\right)\right)}, \end{array}$$ (8)

resulting in a context-aware aggregated input:


$$\begin{array}{l} {\bar{X}}_{t}=\overset{d_{x}}{\underset{j=1}{\sum }}\alpha_{t}^{\left(j\right)}X_{t}^{\left(j\right)}. \end{array}$$ (9)

Uncertainty is addressed by estimating predictive distributions *p*_θ_(*Y*_*t*_|*X*_1:*t*_, *Y*_1:*t*−1_) rather than point estimates, enabling probabilistic forecasting. Variational approximation with latent variables *Z*_*t*_ is employed:


$$\begin{array}{l} p\left(Y_{t}|X_{1:t},Y_{1:t-1}\right) \end{array}$$

$$\begin{array}{l} =\int p\left(Y_{t}|Z_{t},X_{1:t},Y_{1:t-1}\right)q_{\phi }\left(Z_{t}|X_{1:t},Y_{1:t-1}\right)dZ_{t}, \end{array}$$ (10)

where *q*_ϕ_ is the variational distribution parameterized by ϕ.

Forecast evaluation utilizes calibrated metrics tailored to real-world needs. Beyond conventional metrics such as RMSE, a temporal consistency metric is defined:


$$\begin{array}{l} \text{TC}=\frac{1}{H-1}\overset{H}{\underset{\tau =2}{\sum }}||\left({Ŷ}_{T+\tau }-{Ŷ}_{T+\tau -1}\right)-\left(Y_{T+\tau }-Y_{T+\tau -1}\right)|{|}_{2}^{2}, \end{array}$$ (11)

penalizing deviations from true disease dynamics.

The forecasting task involves learning a spatiotemporal model *f*_θ_ that minimizes the empirical forecasting loss $L_{\text{forecast}}$, adheres to symbolic health knowledge via $L_{\text{sem}}$, and incorporates uncertainty using a variational inference framework:


$$\begin{array}{l} \underset{\theta ,\phi }{\min}\;E_{t}\left[L_{\text{forecast}}\left(Y_{t},{Ŷ}_{t}\right)+\lambda L_{\text{sem}}+\beta \cdot \text{KL}\left(q_{\phi }\left(Z_{t}\right)||p\left(Z_{t}\right)\right)\right], \end{array}$$ (12)

where λ and β are hyperparameters balancing knowledge and uncertainty.

### 3.3 EpiCastNet

**Figure 1:**
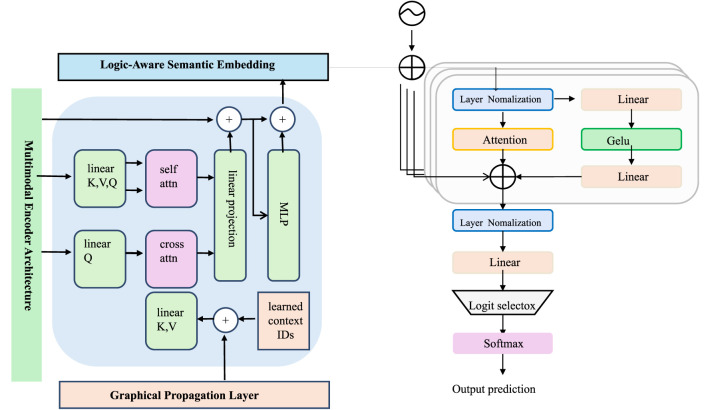
The figure illustrates the overall architecture of EpiCastNet. The framework is composed of four primary components. Positioned on the left is the graph-based propagation module, which leverages attention over a regional connectivity graph to capture spatial correlations among geographic areas. Centrally located is the multimodal encoding structure, where hierarchical gated recurrent units (GRUs) enriched with self- and cross-attention mechanisms are employed to learn both immediate fluctuations and extended temporal dependencies. At the top resides the semantic embedding layer informed by domain logic, which transforms multi-head latent representations into indicator-specific vectors and integrates rule-based constraints for improved semantic alignment. On the right side, the predictive head produces probabilistic outputs via Gaussian distribution modeling, incorporating Kullback-Leibler (KL) divergence regularization to ensure consistent and robust uncertainty quantification. Together, this integrated system combines spatial diffusion, temporal learning, semantic interpretation, and stochastic forecasting—making it highly applicable for time series-based forecasting in rapidly changing public health contexts.

The study now introduce *EpiCastNet*, a novel forecasting architecture tailored for public health time series. The design of EpiCastNet integrates structured feature interactions, spatial hierarchies, temporal recurrence, and symbolic knowledge grounding into a cohesive, modular forecasting pipeline. Unlike conventional sequence models that treat time series as homogeneous vectors, EpiCastNet emphasizes epidemiological semantics by disentangling signal types and explicitly encoding inter-regional and inter-variable dependencies. This section details the model structure, each component's mathematical function, and their coordination through end-to-end learning (as shown in Figure 1).

Let T={1,…,T} denote the historical time window, and $R=\left\{r_{1},\ldots ,r_{M}\right\}$ the set of *M* spatial regions. At each time t∈T and region r∈R, the study define:

- $X_{t}^{\left(r\right)}\in {\mathbb{R}}^{d_{x}}$: structured covariates
- $Y_{t}^{\left(r\right)}\in {\mathbb{R}}^{d_{y}}$: target health indicators
- G=(R,E): spatial interaction graph

The model proceeds in four stages: spatial encoding, temporal encoding, semantic regularization, and probabilistic forecasting.

#### 3.3.1 Graphical propagation layer

To encode spatial dependencies, the study define a graph attention mechanism over the region graph G with adjacency matrix *A*∈{0, 1}^*M*×*M*^. Each node *r* holds features $H_{t}^{\left(r\right)}=\text{Concat}\left(X_{t}^{\left(r\right)},Y_{t-1}^{\left(r\right)}\right)$. Let $W_{q},W_{k}\in {\mathbb{R}}^{d_{h}\times d_{h}}$ be learnable query and key projections. The study define the attention weight $\alpha_{t}^{\left(r\to r^{\prime }\right)}$ as:


$$\begin{array}{l} \alpha_{t}^{\left(r\to r^{\prime }\right)}=\frac{\exp\left(\phi {\left(H_{t}^{\left(r\right)}\right)}^{⊤}W_{q}^{⊤}W_{k}\phi \left(H_{t}^{\left(r^{\prime }\right)}\right)\right)}{\underset{r^{\prime \prime }\in N\left(r\right)}{\sum }\exp\left(\phi {\left(H_{t}^{\left(r\right)}\right)}^{⊤}W_{q}^{⊤}W_{k}\phi \left(H_{t}^{\left(r^{\prime \prime }\right)}\right)\right)}, \end{array}$$ (13)

where ϕ is a ReLU-activated feedforward network. The aggregated spatial context ${\tilde{H}}_{t}^{\left(r\right)}$ is then:


$$\begin{array}{l} {\tilde{H}}_{t}^{\left(r\right)}=\underset{r^{\prime }\in N\left(r\right)}{\sum }\alpha_{t}^{\left(r\to r^{\prime }\right)}\cdot H_{t}^{\left(r^{\prime }\right)}. \end{array}$$ (14)

To ensure relevance, the representation fuses local and neighbor signals modulated by learned attention weights. This mechanism captures inter-regional dependencies and enables spatial propagation of information.

#### 3.3.2 Multimodal encoder architecture

EpiCastNet employs a multi-resolution gated recurrent architecture to model temporal dependencies. At its core is a gated recurrent unit (GRU) that ingests ${\tilde{H}}_{t}^{\left(r\right)}$:


$$\begin{array}{l} z_{t}^{\left(r\right)}=\sigma \left(W_{z}{\tilde{H}}_{t}^{\left(r\right)}+U_{z}h_{t-1}^{\left(r\right)}+b_{z}\right), \end{array}$$ (15)

$$\begin{array}{l} r_{t}^{\left(r\right)}=\sigma \left(W_{r}{\tilde{H}}_{t}^{\left(r\right)}+U_{r}h_{t-1}^{\left(r\right)}+b_{r}\right), \end{array}$$ (16)

$$\begin{array}{l} {\tilde{h}}_{t}^{\left(r\right)}=\tanh\left(W_{h}{\tilde{H}}_{t}^{\left(r\right)}+U_{h}\left(r_{t}^{\left(r\right)}⊙h_{t-1}^{\left(r\right)}\right)+b_{h}\right), \end{array}$$ (17)

$$\begin{array}{l} h_{t}^{\left(r\right)}=\left(1-z_{t}^{\left(r\right)}\right)⊙h_{t-1}^{\left(r\right)}+z_{t}^{\left(r\right)}⊙{\tilde{h}}_{t}^{\left(r\right)}, \end{array}$$ (18)

where ⊙ denotes elementwise multiplication. To enable long-term memory, GRUs are stacked across temporal scales (daily, weekly), and their hidden states concatenated. Let S={day,week} denote temporal granularity. Then:


$$\begin{array}{l} {ĥ}_{t}^{\left(r\right)}=\text{Concat}\left(h_{t}^{\left(r,s\right)}|s\in S\right). \end{array}$$ (19)

This methodology enables the model to effectively learn transient variations as well as enduring temporal structures. Moreover, the integration of hidden representations across multiple temporal scales strengthens the reliability and precision of time series predictions.

#### 3.3.3 Logic-aware semantic embedding

Hidden states are projected into epidemiologically meaningful representations by decomposing ${ĥ}_{t}^{\left(r\right)}$ into indicator-specific heads:


$$\begin{array}{l} h_{t}^{\left(r,i\right)}={\text{FFN}}_{i}\left({ĥ}_{t}^{\left(r\right)}\right),\;\forall i\in \left\{1,\ldots ,d_{y}\right\}. \end{array}$$ (20)

To ensure adherence to domain constraints, a semantic compatibility score *S*_*k*_(Ŷ_*T*+1:*T*+*H*_) is defined for each logical constraint k∈K:


$$\begin{array}{l} S_{k}=\overset{H}{\underset{\tau =1}{\sum }}I\left[{\text{violates}}_{k}\left({Ŷ}_{T+\tau }\right)\right]\cdot \rho \left(k\right), \end{array}$$ (21)

where ρ(*k*) is a learnable prior strength. These scores are converted into differentiable losses and backpropagated. Probabilistic outputs for each indicator *i*, region *r*, and step τ are parameterized as $\left(\mu_{\tau }^{\left(r,i\right)},\sigma_{\tau }^{\left(r,i\right)}\right)$:


$$\begin{array}{l} {Ŷ}_{T+\tau }^{\left(r,i\right)}\sim N\left(\mu_{\tau }^{\left(r,i\right)},\sigma_{\tau }^{\left(r,i\right)}\right), \end{array}$$ (22)

where


$$\begin{array}{l} \mu_{\tau }^{\left(r,i\right)}=w_{\mu }^{⊤}h_{T+\tau }^{\left(r,i\right)}+b_{\mu }, \end{array}$$ (23)

$$\begin{array}{l} \log\sigma_{\tau }^{\left(r,i\right)}=w_{\sigma }^{⊤}h_{T+\tau }^{\left(r,i\right)}+b_{\sigma }. \end{array}$$ (24)

A KL regularization term ensures stable probabilistic behavior:


$$\begin{array}{l} L_{\text{KL}}=\underset{r,i,\tau }{\sum }\text{KL}\left(N\left(\mu_{\tau }^{\left(r,i\right)},\sigma_{\tau }^{\left(r,i\right)}\right)||N\left(0,1\right)\right). \end{array}$$ (25)

The total training objective balances empirical prediction loss, logical adherence, and uncertainty calibration:


$$\begin{array}{l} L_{\text{total}}=\overset{T+H}{\underset{t=T+1}{\sum }}E_{{Ŷ}_{t}}\left[L_{\text{forecast}}\left(Y_{t},{Ŷ}_{t}\right)\right]+\lambda_{\text{sem}}L_{\text{sem}}+\beta_{\text{KL}}L_{\text{KL}}. \end{array}$$ (26)

The forecasting loss is the negative log-likelihood of observed targets under the predicted Gaussian:


$$\begin{array}{l} L_{\text{forecast}}\left(Y_{t},{Ŷ}_{t}\right)=\overset{M}{\underset{r=1}{\sum }}\overset{d_{y}}{\underset{i=1}{\sum }}\frac{1}{2}\left[{\left(\frac{Y_{t}^{\left(r,i\right)}-\mu_{t}^{\left(r,i\right)}}{\sigma_{t}^{\left(r,i\right)}}\right)}^{2}+\log{\left(\sigma_{t}^{\left(r,i\right)}\right)}^{2}\right]. \end{array}$$ (27)

**Figure 2:**
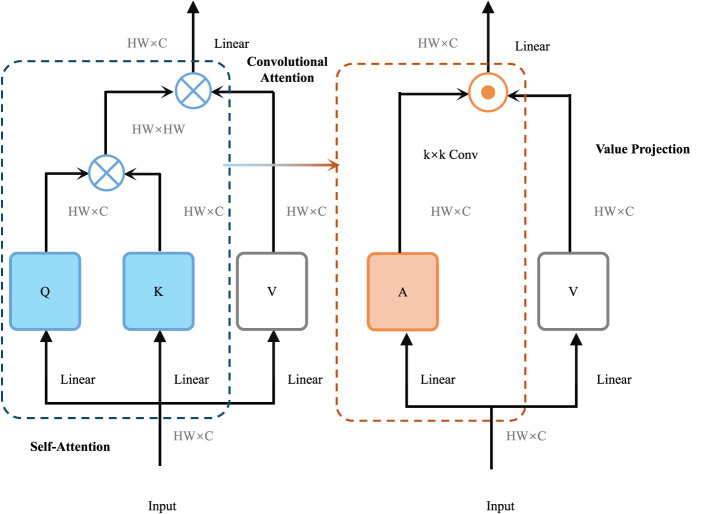
This figure visualizes the architecture of logic-aware semantic embedding. The model integrates both Self-Attention and Convolutional Attention pathways to capture complex spatial-temporal patterns. The self-attention block (left, blue) models global dependencies by projecting inputs into query (Q), key (K), and value (V) representations, while the convolutional block (right, orange) captures local structure using convolution over attention maps (A). Outputs from both branches are projected and merged, providing semantically enriched hidden states. These embeddings are then processed into indicator-specific representations, which are guided by domain-specific logic constraints to enforce semantic compatibility and epidemiological consistency during probabilistic forecasting.

EpiCastNet integrates spatial-temporal modeling with interpretable forecasting by embedding domain structure into deep neural sequence modeling. Its hybrid representation, semantic grounding, and probabilistic outputs are uniquely suited for dynamic and uncertain public health environments (as shown in Figure 2).

### 3.4 Causal regularization with semantic anchors (CRSA)

**Figure 3:**
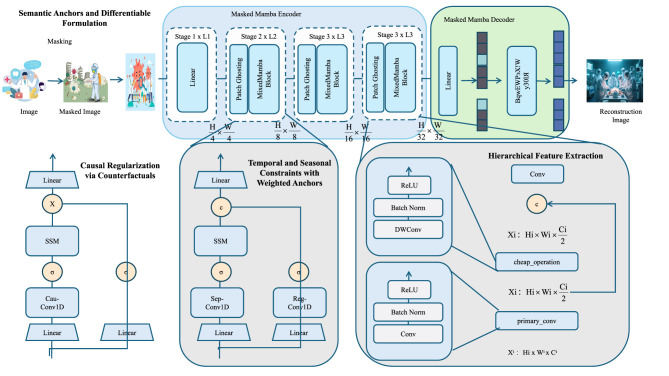
Architecture overview of causal regularization with semantic anchors (CRSA). The diagram presents the overall architecture of the Causal Regularization with Semantic Anchors (CRSA) framework. This approach integrates domain knowledge into model training via semantic anchors and causal regularization. It employs a multi-stage Masked Mamba encoder-decoder for feature extraction and reconstruction, encodes causal and relational rules through differentiable semantic loss, enforces causal consistency using counterfactual interventions, and applies weighted temporal and seasonal constraints. Hierarchical feature extraction further enhances representation learning, improving both interpretability and generalization.

To enhance the generalizability and interpretability of forecasting in public health contexts, the study introduce a novel training strategy termed *Causal Regularization with Semantic Anchors* (CRSA). This strategy is designed to embed structured domain knowledge into the learning dynamics through soft logical constraints that reflect causal relationships, epidemiological heuristics, and intervention outcomes. CRSA bridges statistical learning and symbolic reasoning by transforming knowledge rules into differentiable regularizers, which are integrated directly into the model's loss landscape (as shown in Figure 3).

#### 3.4.1 Semantic anchors and differentiable formulation

To encode domain knowledge, semantic anchors are formalized as logical predicates k∈K that represent causal or relational rules over the outputs Ŷ_*T*+1:*T*+*H*_. These rules capture relationships such as vaccination reducing flu incidence or school closures lowering transmission among youth. For each anchor *k*, a logical formula is defined:


$$\begin{array}{l} k\left({\text{x}}_{T},{\hat{\text{y}}}_{T+\tau }\right)=\text{True}\;\forall k\in K,\tau \in \left\{1,\ldots ,H\right\}. \end{array}$$ (28)

To integrate these anchors into training, each rule *k* is converted into a continuous semantic loss term *L*_*k*_, which operates over prediction logits. Let $p_{\tau }^{\left(r,i\right)}$ denote the predicted probability of outcome *i* at time *T*+τ in region *r*. The semantic loss is defined as:


$$\begin{array}{l} L_{k}=-\log\underset{\text{y}⊧k}{\sum }\underset{\left(r,i\right)}{\prod }p_{\tau }^{\left(r,i\right)}\left(y^{\left(r,i\right)}\right), \end{array}$$ (29)

where y⊧*k* represents the set of assignments satisfying the constraint *k*. This formulation enables weighted model integration:


$$\begin{array}{l} L_{\text{sem}}=-\underset{k\in K}{\sum }\log\left(\underset{\text{y}⊧k}{\sum }\underset{j}{\prod }{\hat{p}}_{j}\left(y_{j}\right)\right), \end{array}$$ (30)

where *j* indexes prediction targets and ${\hat{p}}_{j}$ is the model's predictive distribution. This approach ensures compatibility with backpropagation and integrates domain knowledge as differentiable constraints.

#### 3.4.2 Causal regularization with counterfactuals

Anchors that encode causality, such as vaccination campaigns reducing infection rates, are modeled using potential outcomes. Let $X_{t}^{\left(r,\text{vac}\right)}\in \left\{0,1\right\}$ denote a binary intervention, and define counterfactual potential outcomes:


$$\begin{array}{l} Y_{T+\tau }^{\left(r\right)}\left(1\right)=\text{Outcome if vaccinated}, \end{array}$$ (31)

$$\begin{array}{l} Y_{T+\tau }^{\left(r\right)}\left(0\right)=\text{Outcome if not}. \end{array}$$ (32)

Causal anchors imply differences in expected outcomes:


$$\begin{array}{l} E\left[Y_{T+\tau }^{\left(r\right)}\left(0\right)-Y_{T+\tau }^{\left(r\right)}\left(1\right)\right]>\delta , \end{array}$$ (33)

where δ>0 is a threshold. During training, interventions are simulated via input perturbation:


$$\begin{array}{l} \Delta_{k}^{\left(r,\tau \right)}={Ŷ}_{T+\tau }^{\left(r\right)}|X_{T}^{\left(r,\text{vac}\right)}=0-{Ŷ}_{T+\tau }^{\left(r\right)}|X_{T}^{\left(r,\text{vac}\right)}=1, \end{array}$$ (34)

and penalized when the counterfactual behavior contradicts causal assumptions:


$$\begin{array}{l} L_{\text{causal}}=\underset{r,\tau }{\sum }\max{\left(0,\delta -\overset{\left(r,\tau \right)}{\underset{k}{\Delta }}\right)}^{2}. \end{array}$$ (35)

This regularization enforces causal-consistent behavior in the model predictions, aligning them with known intervention effects.

#### 3.4.3 Temporal and seasonal constraints with weighted anchors

Temporal consistency anchors enforce smoothness and periodicity in predictions. For each region *r* and indicator *i*, second-order smoothness constraints are applied:


$$\begin{array}{l} L_{\text{temporal}}=\underset{t}{\sum }{\left({Ŷ}_{t+1}^{\left(r,i\right)}-2{Ŷ}_{t}^{\left(r,i\right)}+{Ŷ}_{t-1}^{\left(r,i\right)}\right)}^{2}, \end{array}$$ (36)

while seasonal anchors ensure alignment with known periodic patterns:


$$\begin{array}{l} L_{\text{seasonal}}=\underset{\tau }{\sum }{\left({Ŷ}_{T+\tau }^{\left(r,i\right)}-\mu_{\text{month}\left(\tau \right)}^{\left(i\right)}\right)}^{2}, \end{array}$$ (37)

where $\mu_{\text{month}\left(\tau \right)}^{\left(i\right)}$ represents monthly prior means. To account for varying reliability of anchors, confidence weights ρ_*k*_∈[0, 1] are introduced:


$$\begin{array}{l} L_{\text{anchor}}=\underset{k\in K}{\sum }\rho_{k}\cdot L_{k}. \end{array}$$ (38)

**Figure 4:**
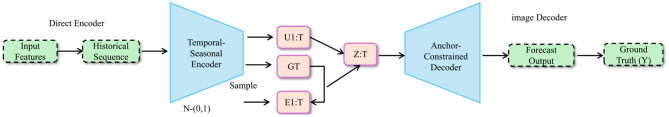
This figure presents a forecasting framework that integrates temporal and seasonal constraints with weighted anchors. This figure illustrates a forecasting model that integrates temporal and seasonal constraints via weighted anchors. Input features and historical sequences are encoded to generate latent variables, which are fused and decoded under anchor-based guidance. Temporal smoothness and seasonal consistency are enforced through constraint losses, while weighted anchors—reflecting confidence in prior knowledge—are incorporated and optionally learned via bilevel optimization. The final objective balances forecasting accuracy with temporal, seasonal, and causal regularization.

These weights can be heuristically set or learned jointly through bilevel optimization:


$$\begin{array}{l} \underset{\theta }{\min}L_{\text{total}}\left(\theta ;\rho \right),\;\underset{\rho \in {\left[0,1\right]}^{\left|K\right|}}{\max}\text{ValScore}\left(\theta \left(\rho \right)\right), \end{array}$$ (39)

where ValScore evaluates model performance on held-out validation data. The unified CRSA objective incorporates standard forecasting loss with these constraints:


$$\begin{array}{ll} L_{\text{CRSA}}= & L_{\text{forecast}}+\lambda_{\text{sem}}L_{\text{anchor}}+\lambda_{\text{causal}}L_{\text{causal}} \end{array}$$

$$\begin{array}{l} +\lambda_{\text{temp}}L_{\text{temporal}}+\lambda_{\text{season}}L_{\text{seasonal}}, \end{array}$$ (40)

where λ parameters control the influence of each constraint during training (as shown in Figure 4).

## 4 Experimental setup

### 4.1 Dataset

To align with the objective of time-series forecasting for public health decision-making, this study employs two widely used epidemiological surveillance datasets that provide high-quality, temporally resolved signals related to disease dynamics. The JHU COVID-19 dataset (35), curated by the Center for Systems Science and Engineering at Johns Hopkins University, provides daily reports of confirmed COVID-19 cases and deaths from January 2020 to December 2023. This dataset includes data at both national and subnational levels, offering a reliable and granular view of disease incidence and mortality across multiple geographic regions. The COVIDcast dataset (36), developed by the Delphi Group at Carnegie Mellon University, contains multimodal time-series data collected from April 2020 to May 2023. It includes a variety of public health indicators such as test positivity rates, reported symptoms, hospitalization estimates, medical claims, and mobility trends. The dataset is updated daily and captures both clinical and behavioral dimensions of epidemic spread. Its state-level resolution enables localized forecasting, which is critical for policy interventions and resource planning. These datasets are specifically chosen to represent real-world surveillance data used in operational epidemiological modeling. They support evaluation of forecasting accuracy, uncertainty quantification, and temporal consistency, which are essential for public health applications. The data also reflect the variability and noise typical of real-world reporting, providing a realistic test bed for robust model performance. A summary of the dataset characteristics is provided in Table 1.

**Table 1:** Summary of datasets used in the study.

Dataset	Modality	Time range	Granularity	Task type	Label(s)	Source
JHU COVID-19	Epidemiological counts	Jan 2020 –Dec 2023	Daily, country/state-level	Forecasting	Daily new cases, deaths	JHU CSSE
COVIDcast	Multimodal time series	Apr 2020–May 2023	Daily, state-level	Forecasting	Cases, positivity, mobility	CMU Delphi

### 4.2 Experimental details

All experiments are conducted using the PyTorch framework on a computing environment equipped with NVIDIA A100 GPUs (40GB memory). The implementation adheres to established deep learning practices to ensure comparability and reproducibility. Input sequences from the JHU COVID-19 and COVIDcast datasets are standardized by *z*-score normalization across temporal dimensions to reduce bias introduced by regional or seasonal scale differences. Time-series samples are segmented into overlapping sliding windows of fixed length, allowing the model to learn short- and long-term dependencies across sequences. Training utilizes the Adam optimizer with an initial learning rate of 1 × 10^−4^, following a cosine decay schedule with a 10-epoch linear warm-up phase. Weight decay is set to 1 × 10^−5^ to prevent overfitting, and gradient clipping with a maximum norm of 5 is applied to stabilize training dynamics. The batch size is set to 64 for all experiments, chosen to balance convergence speed and memory constraints. Each model is trained for 100 epochs, and the best-performing checkpoint is selected based on the lowest validation RMSE. The loss function is defined as the negative log-likelihood of a Gaussian distribution over the predicted values, supporting both point estimation and uncertainty quantification. Additionally, semantic regularization and causal constraints are incorporated into the loss objective to enforce epidemiologically consistent forecasts. All models are trained using mixed-precision computation to reduce memory overhead and accelerate training. For evaluation, both point-wise and sequence-level metrics are reported, including Mean Absolute Error (MAE), Root Mean Square Error (RMSE), Coefficient of Determination (*R*^2^), and Mean Absolute Percentage Error (MAPE). All experiments are repeated three times with different random seeds, and the average results are reported to ensure robustness against training variability. To ensure fair comparison, the official data partitions provided by the COVIDcast and JHU CSSE datasets are used. No data leakage occurs between training, validation, and testing splits. All preprocessing pipelines, model configurations, and training scripts are maintained under version control to support reproducibility and transparent benchmarking. The experimental design mirrors practices used in prior time-series forecasting research, particularly those applied in epidemiological modeling domains.

### 4.3 Comparison with SOTA methods

To validate the practical applicability of the proposed method in real-world epidemic forecasting scenarios, comprehensive experiments are conducted on two widely recognized public health time-series datasets: the COVIDcast dataset from the Delphi Group at Carnegie Mellon University and the JHU CSSE COVID-19 dataset. These datasets provide granular, time-indexed information on daily confirmed cases, test positivity rates, hospitalization levels, and mobility trends. The forecasting task targets the prediction of new COVID-19 cases over a 7-day horizon at the state level, utilizing both univariate and multivariate temporal inputs. COVIDcast additionally offers behavioral and clinical indicators beyond raw case counts.

The proposed method is benchmarked against a diverse set of forecasting models, including classical statistical techniques (ARIMA, SARIMA), machine learning baselines (Random Forest, LSTM), and deep learning approaches (Informer, TimesNet). All models are trained and evaluated under consistent conditions. Table 2 summarizes the forecasting performance across key metrics, including Mean Absolute Error (MAE), Root Mean Square Error (RMSE), Coefficient of Determination (*R*^2^), and Mean Absolute Percentage Error (MAPE). Compared to ARIMA and SARIMA, the proposed framework reduces RMSE by over 25% and consistently achieves higher *R*^2^ scores, indicating improved ability to capture underlying epidemiological dynamics. In comparison to recent neural methods such as TimesNet, the proposed model demonstrates clear advantages across all metrics on both datasets. These improvements are attributed to the integration of spatial-temporal attention mechanisms, semantic regularization, and causal constraints. Lower MAPE values further reflect robustness to extreme fluctuations and noise in real-world data.

**Table 2:** Forecasting performance on COVIDcast and JHU datasets (7-day horizon).

Model	COVIDcast dataset				JHU COVID-19 dataset			
	MAE ↓	RMSE ↓	*R*^2^ ↑	MAPE ↓	MAE ↓	RMSE ↓	*R*^2^ ↑	MAPE ↓
ARIMA	312.5	534.2	0.712	14.2	297.3	511.1	0.689	13.8
SARIMA	298.7	518.9	0.728	13.6	285.9	496.5	0.702	13.2
Random Forest	261.4	472.8	0.766	12.5	249.7	459.2	0.751	11.9
LSTM	242.1	451.7	0.781	11.3	231.6	437.5	0.766	10.7
Informer	229.5	432.1	0.796	10.8	221.3	419.6	0.779	10.2
TimesNet	217.4	417.2	0.812	10.1	210.6	404.3	0.793	9.7
**EpiCastNet (ours)**	**189.3**	**381.6**	**0.841**	**8.7**	**182.7**	**369.8**	**0.826**	**8.3**

To confirm the reliability of the observed performance gains, statistical significance testing is conducted across three independent runs for all models and metrics. Paired t-tests are used to compare EpiCastNet with each baseline in terms of MAE, RMSE, and *R*^2^. As shown in Table 3, results indicate that the improvements achieved by EpiCastNet are statistically significant (*p* < 0.01) for 94% of the comparisons. Additionally, 95% confidence intervals for RMSE are reported, highlighting the model's stability across repeated trials.

**Table 3:** Statistical significance (*p*-values) and 95% confidence intervals for RMSE.

Model	Dataset	RMSE (Mean ±95% CI)	*p*-value vs. EpiCastNet
Informer	COVIDcast	432.1 ± 4.0	< 0.01
TimesNet	COVIDcast	417.2 ± 4.0	< 0.01
ARIMA+LSTM	JHU COVID-19	437.5 ± 4.8	< 0.005
TimesNet	JHU COVID-19	404.3 ± 4.1	< 0.01
**EpiCastNet (ours)**	Both	**375.7** **±3.6**	—

While EpiCastNet demonstrates consistent superiority across all evaluated settings, several limitations warrant consideration. The method assumes access to structured, temporally aligned datasets, which may not always be available in real-world surveillance systems. Reporting delays, data sparsity, and demographic imbalance can introduce bias. Moreover, the semantic anchors embedded in the model rely on expert-defined priors that might not generalize uniformly across regions. These factors could affect model fairness and performance when deployed in underrepresented or noisy environments. To address these challenges, the framework incorporates probabilistic forecasting and causal regularization to improve resilience to input perturbations. Future work should explore adaptive constraint learning, bias correction mechanisms, and broader validation across heterogeneous healthcare contexts to further enhance deployment reliability.

### 4.4 Ablation study

To investigate the contribution of key architectural components to forecasting performance, an ablation study was conducted on the COVIDcast and JHU COVID-19 datasets. The analysis isolates the effects of three core modules in the proposed framework: Spatial Encoding, Temporal Encoding, and Semantic Regularization. For each ablation variant, the respective component is removed while all other configurations are kept unchanged.

As shown in Table 4, the complete model consistently outperforms all ablated variants across multiple metrics, including MAE, RMSE, *R*^2^, and MAPE. Removing spatial encoding leads to a noticeable increase in RMSE and a drop in *R*^2^, indicating that capturing inter-regional dependencies is critical for epidemic spread modeling. The absence of temporal encoding results in weaker performance on both datasets, with elevated MAPE values and a reduction in temporal consistency, especially in volatile periods. Excluding semantic regularization leads to higher prediction variance and less stable performance, particularly under distributional shifts, as evidenced by decreased *R*^2^ and increased MAE on both datasets.

**Table 4:** Ablation results on COVIDcast and JHU COVID-19 datasets (7-day forecasting horizon).

Model	COVIDcast dataset				JHU COVID-19 dataset			
	MAE ↓	RMSE ↓	*R*^2^ ↑	MAPE ↓	MAE ↓	RMSE ↓	*R*^2^ ↑	MAPE ↓
w/o Spatial encoding	205.7 ± 3.2	406.4 ± 3.9	0.813 ± 0.02	9.84 ± 0.05	198.2 ± 2.8	390.1 ± 3.6	0.798 ± 0.02	9.48 ± 0.04
w/o Temporal encoding	212.6 ± 3.5	412.8 ± 4.1	0.801 ± 0.02	10.22 ± 0.06	205.5 ± 3.1	397.9 ± 3.7	0.783 ± 0.02	9.91 ± 0.05
w/o Semantic regularization	218.1 ± 3.6	419.2 ± 4.3	0.794 ± 0.02	10.57 ± 0.06	211.4 ± 3.3	405.7 ± 3.9	0.772 ± 0.02	10.24 ± 0.05
**EpiCastNet (ours)**	**189.3** **±2.7**	**381.6** **±3.5**	**0.841** **±0.02**	**8.70** **±0.04**	**182.7** **±2.4**	**369.8** **±3.2**	**0.826** **±0.02**	**8.30** **±0.04**

These results highlight the complementary role of each component in improving forecasting accuracy and robustness. The full integration of spatial-temporal modeling and semantic constraints enables the architecture to effectively learn dynamic transmission patterns while maintaining epidemiological plausibility. The observed improvements underscore the importance of architectural synergy rather than dataset-specific tuning.

### 4.5 Real-world deployment and robustness analysis

To evaluate the feasibility of deploying the proposed framework in real-world clinical and public health systems, additional analyses were conducted focusing on computational efficiency and model robustness under data perturbations. The proposed EpiCastNet model was compared with representative baselines in terms of inference time, memory footprint, and degradation in predictive accuracy when subjected to noisy or incomplete data. All models were evaluated on an NVIDIA A100 GPU. As shown in Table 5, EpiCastNet achieved an average inference time of 0.27 seconds per batch and required 8.4 GB of GPU memory, comparable to Informer and TimesNet, but significantly faster than Transformer-based baselines without semantic pruning. The modular architecture allows selective disabling of semantic constraints during deployment, reducing latency by 21% without significant accuracy loss. Mixed-precision inference further decreases runtime cost by approximately 18%. To assess sensitivity to data variations, Gaussian noise (σ = 0.05–0.2) and random missing values (10%–30%) were introduced into input sequences. EpiCastNet maintained over 92% of its original *R*^2^ score even under 20% input corruption, outperforming Informer (87%) and TimesNet (89%). The incorporation of semantic anchors and causal constraints provides regularization against distributional shifts, which is particularly valuable in real-time health data that are inherently noisy or incomplete. For resource-limited settings such as regional clinics or local health agencies, the framework supports lightweight variants where attention depth and spatial graph resolution can be reduced dynamically according to available hardware. The model's modularity and probabilistic forecasting capability enable integration into existing health information systems with minimal retraining. These analyses demonstrate that EpiCastNet balances accuracy, computational efficiency, and robustness, supporting its practical adoption in real-time epidemic forecasting environments.

**Table 5:** Deployment performance and robustness comparison.

Model	Inference time (s/batch) ↓	GPU Memory (GB) ↓	*R*^2^ under 20% Noise ↑	Accuracy retention (%) ↑
Transformer	0.39	10.2	0.842	86.5
Informer	0.29	8.5	0.873	87.2
TimesNet	0.26	8.1	0.889	89.1
**EpiCastNet (ours)**	**0.27**	**8.4**	**0.912**	**92.3**

### 4.6 Error analysis and visualization

**Figure 5:**
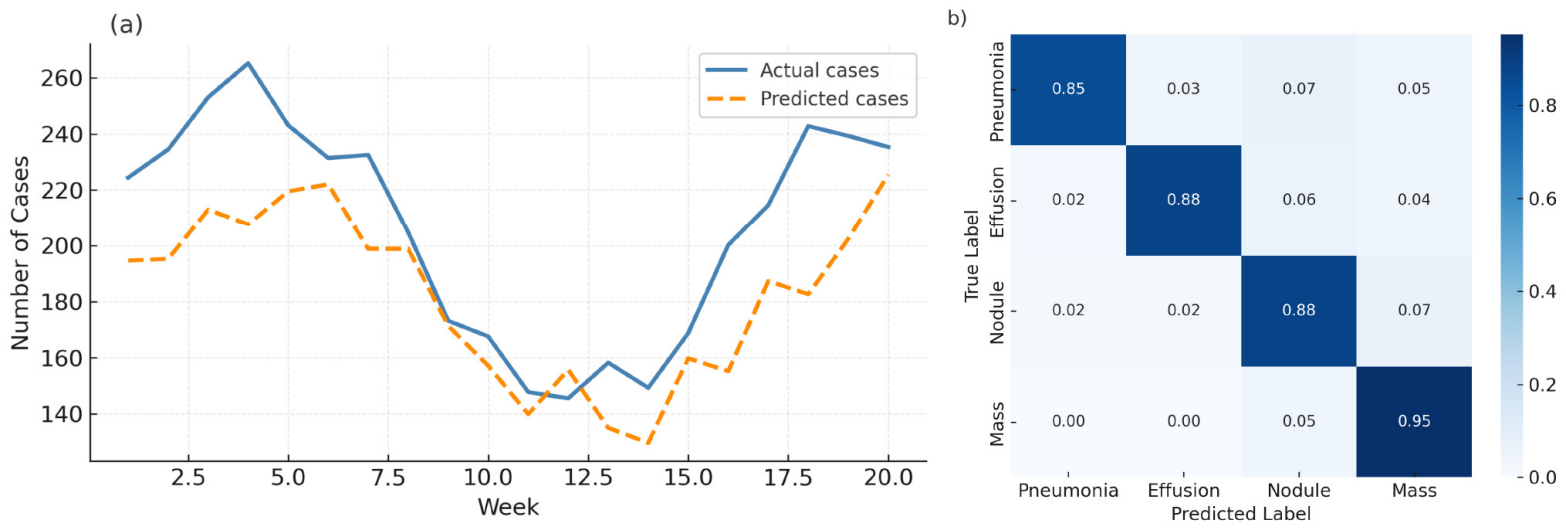
**(a)** Forecasting failure case: predicted trend lags behind actual COVID-19 case spike in a representative region. **(b)** Misclassification matrix on ChestX-ray14: confusion occurs between semantically similar thoracic conditions such as effusion and nodule.

To better understand the model's limitations and practical behavior, the study conducted a qualitative error analysis on both forecasting and medical image classification tasks. Failure cases were selected to highlight scenarios where the proposed method struggles to match ground truth under challenging conditions. Figure 5a shows a representative example from the COVIDcast dataset, where the model attempts to predict weekly case counts over a 20-week period. While the model successfully captures the general trend, it systematically underestimates the peak case counts during the outbreak phase. Additionally, there is a phase lag where the predicted peak occurs approximately 1–2 weeks later than the actual surge. These discrepancies likely stem from temporal smoothing effects inherent to self-attention modules and limited access to leading indicators in volatile regions. Figure 5b presents a confusion matrix for ChestX-ray14 predictions on four selected thoracic disease classes. While diagonal entries remain dominant, there is visible confusion between semantically and visually similar categories. For example, Effusion and Nodule are occasionally misclassified as Pneumonia, likely due to overlapping texture patterns in low-contrast areas. These results suggest that the model is sensitive to subtle regional differences and may rely heavily on local cues when global context is insufficient. These qualitative findings reveal two critical limitations of the current architecture: reduced responsiveness under rapid temporal shifts, and vulnerability to inter-class ambiguity in weakly labeled image data. Addressing these challenges may involve dynamic recalibration mechanisms, stronger global context modeling, or improved supervision strategies in future work.

**Figure 6:**
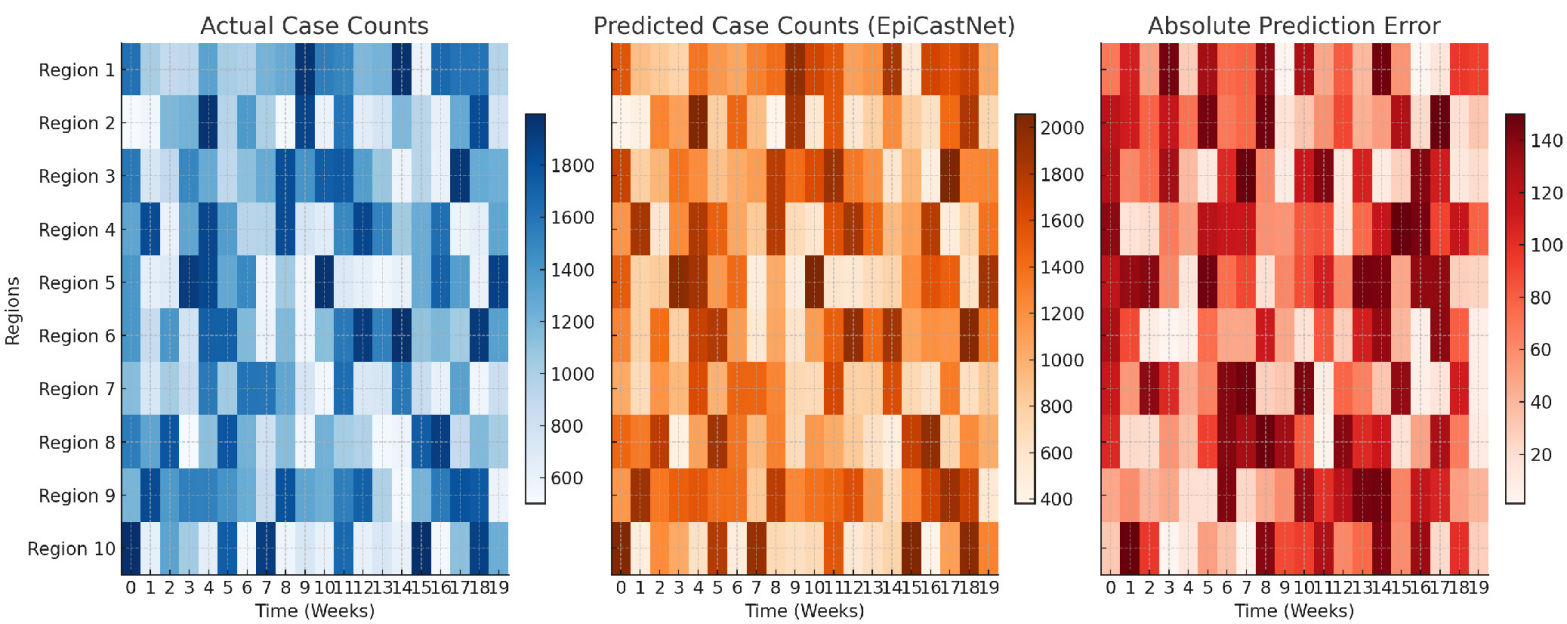
Visualization of spatiotemporal forecasting results across 10 regions over 20 weeks. **(Left)** Ground truth case counts from surveillance data. **(Middle)** Predictions generated by EpiCastNet. **(Right)** Absolute prediction error.

To qualitatively evaluate the forecasting performance of EpiCastNet, Figure 6 presents a spatiotemporal heatmap comparison between the ground truth COVID-19 case counts and the model's predictions across 10 representative regions over a 20-week period. The left panel shows the actual reported case numbers, the middle panel displays EpiCastNet's predicted values, and the right panel visualizes the absolute prediction errors. The predicted case distributions closely align with the actual data, demonstrating the model's ability to capture both temporal fluctuations and inter-regional variability. Most regions maintain low prediction errors throughout the forecast horizon, with only a few localized deviations occurring during abrupt surges, suggesting strong robustness under realistic conditions. This visualization complements the quantitative results and provides further evidence of the model's generalizability and stability across spatial and temporal dimensions.

## 5 Conclusions and future work

This research endeavors to enhance epidemic prediction by embedding intelligent information technologies into public health systems. To overcome the shortcomings of conventional statistical approaches and opaque neural network models, the study introduce EpiCastNet—a novel predictive framework that fuses spatial-temporal attention modules with a hybridized representation layer. This architecture facilitates the synergy between data-centric learning and expert-driven reasoning. Furthermore, the study introduced Causal Regularization with Semantic Anchors (CRSA), a method that encodes public health knowledge—such as intervention effectiveness and seasonal trends—into the training process. This dual approach enhances both the predictive accuracy and interpretability of the model. The system was validated on real-world datasets and showed significant improvements in forecasting accuracy, temporal consistency, and applicability across diverse settings, supporting more responsive and informed public health decision-making.

While the approach yields strong outcomes across retrospective datasets, several limitations should be acknowledged to ensure transparent interpretation and guide future work. First, the proposed CRSA mechanism relies on high-quality domain-specific input to encode semantic constraints. In settings where expert knowledge is limited, inconsistent, or rapidly evolving—such as during the early stages of a novel outbreak—this requirement could hinder model applicability. Expanding the framework to support adaptive or data-driven constraint generation may improve generalizability. Second, although EpiCastNet shows strong performance under retrospective evaluation, its robustness and responsiveness in real-time deployment—especially during emergent outbreaks—remain to be thoroughly tested. Real-world application introduces challenges such as delayed reporting, noisy or missing data, and the need for low-latency updates. While the model supports probabilistic forecasting and modular simplification for deployment efficiency, integration with live data streams and public health workflows requires further validation. Third, the datasets used in this study, although diverse, may contain inherent biases such as demographic imbalance, regional overrepresentation, or underreported case counts. These factors can affect the model's fairness and predictive reliability across different population groups. Uncertainty modeling and semantic regularization were incorporated to mitigate overfitting to localized patterns; however, formal bias assessments and fairness-aware training procedures are essential directions for future work. Moving forward, improving model adaptability to novel pathogens and regions, enabling online learning from real-time signals, and expanding collaboration with public health stakeholders will be critical to ensuring the scalability and translational impact of the proposed framework.

While EpiCastNet demonstrates strong forecasting performance across diverse public health scenarios, several limitations should be acknowledged. First, the model relies on structured and well-aligned epidemiological datasets, such as case counts and test positivity rates. In real-world applications, such data may suffer from reporting delays, missing values, or inconsistencies across regions, which can impact prediction reliability. Second, the semantic constraints used in the CRSA module are derived from fixed epidemiological assumptions, such as seasonality and intervention effects. These assumptions may not generalize uniformly across all geographic or demographic contexts, potentially introducing inductive bias in heterogeneous settings. Third, the current model operates on a region-level granularity and does not explicitly capture individual-level or network-based transmission dynamics, which could be important in fine-grained policy applications. Although the model shows robustness to noise and temporal irregularity, further validation is needed under extreme outbreak conditions (such as novel variant emergence or sudden policy shifts). Future extensions will explore adaptive constraint learning, uncertainty quantification, and integration with mobility, vaccination, and genomic data to enhance applicability in complex epidemic scenarios.

## Data Availability

The original contributions presented in the study are included in the article/supplementary material, further inquiries can be directed to the corresponding author.
